# Needs and Problems of the Caregivers of Psychiatric Patients With Violent Behaviours

**DOI:** 10.7759/cureus.57228

**Published:** 2024-03-29

**Authors:** Anusuya SP, Umadevi A, Sumathy K

**Affiliations:** 1 Nursing/Psychiatric Nursing, Mary Lott Lyles Hospital, Madanapalle, IND; 2 Women Studies, Avinashilingam Institute for Home Science and Higher Education for Women, Coimbatore, IND

**Keywords:** psychiatric patients, violent patient, violent behaviors, problems, needs, caregivers

## Abstract

Background: Violent behaviour exhibited by psychiatric patients is a major problem faced by their family members. Agitated violent patients constitute a part of all emergency psychiatry treatment, and family members carry out most of the care for the mentally ill. Caring for the mentally ill is a burden for the caregivers, and they face difficulties and problems that affect their health and well-being.

Aim: This study aimed to assess the needs and problems of the caregivers of psychiatric patients with violent behaviour.

Method: This study was conducted at Mary Lott Lyles Hospital, Andhra Pradesh, India. Fifty caregivers of patients with violent behaviour were assessed concerning their needs and problems, which were explored using an open-ended questionnaire, and responses were documented and voice-recorded. Qualitative and quantitative analyses were done.

Results: Caregivers of psychiatric patients exhibiting violent behaviour face pressing needs and formidable challenges. They urgently require prompt treatment, detailed explanations from healthcare professionals and hope for their loved ones' full recovery. However, they grapple with managing the unpredictable and aggressive behaviour of patients, transportation difficulties and the pervasive stigma of mental illness. Economic crises further exacerbate their struggles, making it challenging to access necessary care and support for their relatives.

Conclusion: Despite the challenges encountered in handling violent behaviour, caregivers consistently ensured that patients received emergency treatment and ongoing care. They stressed the importance of healthcare professionals understanding their needs and those of the patients. These results highlight the necessity of addressing the comprehensive requirements of both patients and caregivers when dealing with violent behaviour in psychiatric settings.

## Introduction

Violent behaviour exhibited by psychiatric patients is a significant problem faced by mental health team members in their day-to-day services [1]. Agitated (or) violent patients constitute 10% of all emergency psychiatric treatment as most of these people have severe psychiatric problems, such as schizophrenia, affective disorder (or) substance abuse and less frequently organic illness (or) psychological stress that underlines the aggression [2,3].

Violent patients are not a homogenous group, and their violence reflects various biological, psychodynamic and social factors. Most researchers and clinicians agree that a combination of factors plays a role in violence and aggression, although there are differing opinions regarding the importance of individual factors [4].

Mental illness is widely prevalent in India, and the prevalence is certainly not less than what is reported in Western countries. Furthermore, the figure for violence in India is as high in rural as in urban areas. Epidemiological studies report prevalence rates for psychiatric disorders varying from 9.5 to 370 per 1000 people in India. This prevalence encompasses a broad spectrum of mental health disorders, reflecting the diverse challenges individuals face in the country [5]. In India, it is commonly observed that individuals often resort to general health services, including medical outpatient departments, private clinics or primary healthcare centres, seeking assistance for emotional concerns that manifest as physical symptoms.

The public perception of violence is common in psychiatric patients and is evidenced by numerous surveys conducted worldwide. Persons with schizophrenia are thought to be at increased risk of committing violent crimes four to six times the level of general population individuals without this disorder [6].

A 1993 survey by Clements reported that more than half the people agreed with the statement that ‘those with mental disorders are more likely to commit acts of violence' [7]. As mentioned in multiple studies, the major cause of this stigma is the perception that some individuals with mental illnesses are dangerous [7]. Given this fact, it seems self-evident that the stigma will not be decreased until we decrease violent behaviour committed by mentally ill persons [7]. One study found that 61% of adults believed that an individual with schizophrenia was somehow likely to be violent towards others [8].

Violence rates in acute psychiatric wards across high-income countries are disturbingly high and vary significantly between settings. Environments with more patients exhibiting violence risk factors tend to report higher incidents. While traditional risk factors like male gender and alcohol use are significant, modifiable factors within the wards also play an important role. Understanding and measuring violence rates are crucial for developing strategies to protect both patients and staff from such incidents [9]. Younger age, learning difficulties and a history of violence, drug misuse and victimisation predict violent behaviour in psychotic patients [10]. The result of these surveys highlights the magnitude of the problem.

The role of the family may have undergone a transition from being viewed as a possible etiological factor to being a powerful ally in treatment. Individuals and their families already carry out most of the care provided in terms of mental illness. They need help to do this more effectively [11,12].

Family members often witness firsthand the challenging behaviours that can arise in individuals with certain mental illnesses, such as unusual actions, neglect of self-care, self-harming tendencies or thoughts of suicide. These family members may also find themselves on the receiving end of aggression or hostility from individuals they deeply care about. Some family members struggle to come to terms with the fact that their loved ones are grappling with mental illness and may hold unrealistic expectations about how their loved ones should feel or behave [2]. Over time, there has been a growing acknowledgement and documentation of the 'burden of care', which refers to the impact of a family member's mental illness on the caregiver's physical and emotional well-being, as well as their overall quality of life [13,14,2].

A study was done on the rapid tranquillisation of violent or agitated patients in a psychiatric emergency setting. A pragmatic randomised trial of intramuscular lorazepam versus haloperidol plus promethazine, conducted in the department of psychiatry at Christian Medical College in Vellore, stated that prevalence data for violence among people with psychiatric disorders in low- and middle-income countries are scant. There is no evidence to suggest that the prevalence of violent (or) agitated behaviour is rarely any less in low-income countries such as India than elsewhere. Considering the magnitude of the population of India, the magnitude of the problem found by emergency services in India is therefore readily apparent [15]. There has been relatively little research conducted in India on the caregivers of patients with violent behaviour, posing a significant challenge. This lack of exploration prompted us to prioritise this aspect, uncovering caregivers' needs, challenges and perspectives in dealing with such patients. We aim to thoroughly investigate this overlooked area and provide additional insights based on the findings, thus addressing the caregivers' needs and the problems they have experienced.

## Materials and methods

This study was conducted at Mary Lott Lyles Hospital, Andhra Pradesh, India. A descriptive design with a qualitative approach was employed to assess the needs and problems of caregivers of psychiatric patients exhibiting violent behaviour. Fifty caregivers accompanying patients with violent behaviour seeking emergency management at the hospital were selected through consecutive sampling [16]. Caregivers of psychiatric outpatients displaying violent behaviour and those above the age of 18 years were included as study participants. Conversely, caregivers unwilling to participate or those experiencing more than one episode of violent behaviour during the study period were excluded.

Instruments

The Clinical Global Impression (CGI) scale was utilised to assess the severity of the patient's illnesses [17], and the Overt Aggression Scale (OAS) was employed to assess the level of aggression in violent patients [18]. The intraclass correlation coefficient of reliability obtained indicated good reliability (>0.75) for the OAS. In addition, demographic data of both patients and caregivers were collected, along with the needs and problems of caregivers using an explorative questionnaire developed by the investigator. Instruments underwent reliability and validity checks through expert opinions and pilot studies.

Procedure

Every consecutive patient presenting to the outpatient department with violent behaviour underwent an assessment. The clinician utilized the CGI scale to assess illness severity, followed by further assessment by the investigator using the OAS [17,18]. Caregivers of these patients were identified and, upon meeting the inclusion criteria and providing consent, were included in the study. A strong rapport was established with caregivers, and after collecting demographic and clinical data, the investigator spent time listening to their needs and problems, gradually introducing questions. When caregivers encountered difficulty understanding the questionnaire, assistance was provided by the investigator. Responses to explorative questions were recorded with caregivers' permission, ensuring confidentiality.

Data analysis

Descriptive statistics, such as frequency and percentage, were employed to describe caregivers' needs and problems. Audio-recorded interviews were transcribed verbatim, and common and exceptional needs and problems were coded. The frequency percentage of their needs and problems was determined, and the chi-square test was utilized to explore associations between selected demographic variables of caregivers and their needs and problems.

## Results

Demographic characteristics of patients with violent behaviour

This study encompassed 50 patients, chosen through consecutive sampling, who were included based on their manifestation of violent behaviour upon presentation to the emergency department. The analysed data are presented in Table 1. Regarding their demographic information, the majority (n = 32, 64%) of the patients fell within the age group of 19 to 35 years, with 28 (56%) males and 22 (44%) females, and 26 (52%) are married. Furthermore, 37 (74%) of the patients had middle school and above education levels, only two (4%) were categorized as professionals, and 21 (42%) had monthly income below 1000. In addition, 38 (76%) of them are not working at present (Table 1).

**Table 1:** Distribution of the patients in relation to their demographic data. Data are presented as N and %.

Parameter	Category	N	%
Age in years	Adolescents (13-18)	3	6
	Young adult (19-35)	32	64
	Middle adult (36-65)	15	30
Gender	Male	28	56
	Female	22	44
Religion	Hindu	43	86
	Muslim	2	4
	Christian	5	10
Marital status	Single	26	52
	Married	21	42
	Widow(er)	2	4
	Divorced	1	2
Education	Illiterate	7	14
	Primary	6	12
	Middle school	8	16
	Secondary	11	22
	Higher secondary school	8	16
	College	10	20
Occupation	Farmer	7	14
	Labourer	18	36
	Technical job	4	8
	Professional	2	4
	Unskilled	7	14
	Others	12	24
Monthly income (Rs)	<1000	21	42
	1001–3000	20	40
	3001–5000	6	12
	>5000	3	6
Current working status	Yes	12	24
	No	38	76

Demographic characteristics of caregivers of patients with violent behaviour

A total of 50 caregivers participated in this study. The majority (n = 26, 52%) were parents of the patients. In addition, most (n = 32, 64%) belonged to the middle age group (36-65 years), with 28 (56%) of the samples being female. Twenty (40%) caregivers were illiterate, while another 20 (40%) were labourers. Concerning their place of residence, the majority (n = 36, 72%) hailed from rural areas. Furthermore, nine (18%) of the caregivers travelled a distance of 100-200 km to reach the hospital, with the majority (n = 26, 52%) utilizing bus transport (Table 2).

**Table 2:** Distribution of caregivers in relation to their demographic data Data are presented as N and %.

Parameter	Category	N	%
Relationship of the primary informant	Father	9	18
	Mother	17	34
	Husband	5	10
	Wife	6	12
	Brother	8	16
	Sister	3	6
	Son	1	2
	Aunt	1	2
Age (in years)	Young adult (19-35)	14	28
	Middle adult (36-65)	32	64
	Late adult (>65)	4	8
Gender	Male	22	44
	Female	28	56
Marital status	Single	4	8
	Married	40	80
	Widow(er)	6	12
Education	Illiterate	20	40
	Primary	9	18
	Middle school	6	12
	Secondary	7	14
	Higher secondary school	4	8
	College	4	8
Occupation	Farmer	4	8
	Labourer	20	40
	Technical job	1	2
	Professional	4	8
	Unskilled	2	4
	Others	19	38
Place	Urban	6	12
	Semi-urban	8	16
	Rural	36	72
Distance(km)	1-30	16	32
	31-50	10	20
	51-100	14	28
	101-200	9	18
	> 200	1	2
Mode of transport	Bus	26	52
	Auto	11	22
	Car/van	9	18
	Others	4	8

Clinical data of patients and caregivers

The clinical data of the 50 patients brought to the emergency departments show that the majority (n = 40, 80%), were not restrained upon arrival. Most (n = 34, 68%) were accompanied by one to four caregivers, predominantly immediate and close relatives, accounting for 34 (68%) of the cases. Concerning injuries, 35 (70%) patients and an equal number of caregivers did not sustain any injuries. Of the patients, 17 (34%) were diagnosed as manic, while 15 (30%) were diagnosed with schizophrenia. Based on the OAS assessment, the patients were categorized as follows: those with no aggression and mild aggression accounted for seven (14%) of the sample, while those categorized as aggressive, very aggressive and extremely aggressive represented 43 (86%) of the sample (Table 3).

**Table 3:** Clinical data of the patients and caregivers CGI = Clinical Global Impression. Data are presented as N and %.

Parameter	Category	N	%
Restraints at arrival	Yes	10	20
	No	40	80
No. of attendants with patients	01-Apr	34	68
	05-Aug	15	30
	> 8	1	2
Relationship of attendants (other than primary caregivers) with patients	Immediate relative	18	36
	Immediate, close and others	10	20
	Immediate and close	16	32
	Immediate and others	6	12
Any injury to the patient	Yes	15	30
	No	35	70
Any injury to relatives	Yes	15	30
	No	35	70
Any emergency treatment in the previous week	Yes	10	20
	No	40	80
Diagnosis	Schizophrenia	15	30
	Acute psychosis	11	22
	Mania	17	34
	Alcoholic psychosis	1	2
	Others	6	12
Duration of present symptoms	<15 days	34	68
	16-29 days	4	8
	1 month to 6 months	12	24
First episode	Yes	21	42
	No	29	58
Associated illness	Yes	4	8
	No	46	92
Severity of illness (CGI score)	Mildly ill	2	4
	Moderately	19	38
	Markedly ill	22	44
	Severity ill	7	14
Overt Aggression Scale (OAS Score)	Mild aggressive(5to7)	7	14
	Aggressive (8 to 10)	28	56
	Very aggressive (11 to 13)	14	28
	Extremely aggressive (>13)	1	2

Reason for bringing the patient to the hospital

From the caregivers' verbatim, themes related to reasons for bringing the patients were identified and coded. The identified themes were analysed for frequency and percentage. All 50 caregivers (100%) reported a significant change in the patient's behaviour; these data were extracted from the caregivers verbatim, with 30 (60%) expressing challenges in managing the patient at home due to instances of shouting and aggressive behaviour. In addition, two patients (4%) exhibited extreme behaviours, such as fire-setting (Table 4).

**Table 4:** Reason for bringing the patient to the hospital It is shown that each patient had multiple reasons for coming to the hospital. The data presented in each row are calculated based on the total number of patients (N = 50). Data are presented as N and %.

Reasons	N	%
Shouting	30	60
Beating relatives and others	30	60
Not sleeping	24	48
Not eating	16	32
Getting angrier	13	26
Running and wandering	12	24
Not taking medicine	7	14
Fighting	7	14
Over-religious	6	12
Suspicious	6	12
Drinking more alcohol	3	6
Demanding for money	2	4
Fire setting	2	4

Types of violent behaviour exhibited by the patients

All caregivers (N = 50, 100%) reported a significant change in the patient's behaviour. These data were extracted and transcribed from the caregivers' verbatim, with 43 (86%) reporting Verbal abuse, followed by physical aggression (N =41, 82%) and threatening behaviour towards the caregivers and others (N = 39, 78%). In addition, 24 (48%) reported instances where household items were destroyed (Table 5).

**Table 5:** Violent behaviour exhibited by the patients This table indicates that the patients had more than one type of aggressive behaviour. The data presented in each row are calculated based on the total number of caregivers (N = 50). Data are presented as N and %.

Character	N	%
Verbal abuse	43	86
Physical aggression	41	82
Threatening	39	78
Destruction of property	24	48

Caregiver problems concerning the violent behaviour of the patient

From the caregivers' verbatim, themes related to their needs and problems were coded. The identified themes were analysed for frequency and percentage. Caregivers expressed extremely upset (N = 43, 86%) indicating such feelings, and the majority of them cried. In addition, 27 (54%) caregivers reported being frightened by the patient's behaviour, while 13 (26%) expressed fear for their safety. Furthermore, 29 (58%) caregivers felt embarrassed. All 50 (100%) caregivers faced difficulties in bringing the patient to the hospital (Table 6).

**Table 6:** Caregivers’ problems with the violent behaviour of the patient Data are presented as N and %.

Problems expressed by the caregivers	Category	N	%
Upset by the patient's behaviour	Extremely upset	43	86
	Very upset	5	10
	Not upset	2	4
Frightened by their patient’s behaviour	Yes	27	54
	No	23	46
Fear of safety	Yes	13	26
	No	37	74
Embarrassed by the patient's behaviour	Not embarrassed	21	42
	Talking foul language	16	32
	Disrobing behaviour	7	14
	Suspicious talk	6	12
Difficulties in bringing the patient to the hospital	The unwillingness of the patient to come to the hospital	23	46
	Rejected by the public transport	1	2
	No money to bring the patient	9	18
	The caregivers were troubled by the patients while coming in the vehicle to the hospital	17	34
Mental illness known to others	Yes	44	88
	No	6	12

Association of demographic data and needs and problems of the caregivers

It is seen that 22 (100%) of the male caregivers felt that the mental illness was known to others, while seven (25%) of the female caregivers did not feel so. When income was <3000, caregivers were extremely upset about the patient's violent behaviour. These differences were statistically significant (Table 7).

**Table 7:** Association of demographic data of caregivers with needs and problems * Significant

Caregiver sex	Mental illness of the patient known to others				X^2^	df	p-Value
	Yes		No				
	N	%	N	%			
Male	22	100	0	0	4.487	1	0.011*
Female	21	75	7	25			
Income of the caregiver	Upset				20.583	9	0.015*
	Not upset		Extremely upset				
<3000	1	2.439	40	97.55			
3000-5000	1	16.66	5	83.33			
>5000	2	66.66	1	33.33			

Relationship between locational background and problems of caregivers 

Significantly more of the rural caregivers were afraid of their patient's violent behaviour, while the semi-urban (N = 7, 87.5%) and urban (N = 4, 66.66%) were not. Here too, significantly more of the rural caregivers were embarrassed while the majority of semi-urban and urban caregivers were not. In rural places, the illness of the patient was known to others (N = 35, 97.22%) in the majority of the cases (Table 8).

**Table 8:** Relationship between locational background and problems of caregivers * Significant

Caregivers' problems		Location of the caregivers						X^2^	df	p-value
		Urban		Semi-Urban		Rural				
		N	%	N	%	N	%			
Afraid	Yes	2	33.33	1	12.5	24	66.66	8.904	2	0.012*
	No	4	66.66	7	87.5	12	33.33			
Embarrassment	Yes	1	16.33	3	37.5	25	69.44	7.524	2	0.023*
	No	5	83.33	5	62.5	11	30.55			
Illness known to others	Yes	4	66,66	4	50	35	97.22	14.24	2	0.001*
	No	2	33.33	4	50	1	2.77			

## Discussion

Family members who care for patients exhibiting violent behaviour face tremendous burdens. Caregivers also face the very real risk of violence. The American Psychiatric Association reported that family members (65%) are more frequently affected by the violent behaviour of the mentally ill person, and it was found that the violent behaviour was severe before their admission to a psychiatric care facility [19]. During this study, the investigator found that the caregivers and other family members were eager to communicate with nursing staff and other healthcare professionals to ascertain the state of their patients and to express their feelings and problems. 

Data revealed that most of the patients with violent behaviour (N = 32, 64%) were young adults (19-34 years) and males (N = 28, 56%). Regarding diagnosis, schizophrenia was diagnosed in 15 (30%), while acute psychosis was diagnosed in 11 (22%). This sample resembles instances of aggressive patients documented in the literature. In other series, individuals prone to violence were found to have a higher likelihood of being diagnosed primarily with schizophrenia and displaying a greater tendency towards experiencing hallucinations; most of the assaults were committed by young adults and serious assaults were committed by men [20,21]. Among the caregivers, 26 (52%) were parents, and the remaining (N = 24, 48%) of them were immediate and close relatives, while 29 (58%) of the patients had exhibited repeated attacks of violent behaviour, which support the literature that violent behaviour is often repetitive [20,21].

Caregivers expressed their needs and problems concerning the violent behaviour of the patient, and thematic analysis revealed that caregivers (N = 50, 100%) brought the patient to the hospital since there was a behaviour change, which they could not manage at home (N = 32, 64%). The mean number of days that the patient showed violent behaviour before coming to the hospital was six days.

Furthermore, caregivers also expressed their difficulties. The majority (N = 44, 88%) of the caregivers reported being verbally abused (N = 41, 82%) were physically abused (hit, bitten, and beaten) or subjected to threatening behaviour (N = 39, 78%). This is consistent with a previous study on violence by psychiatric in-patients, where the majority of patients were admitted with complaints of damage to property, verbal aggression, physical aggression against family members and others and threatening behaviour [21]. Of all caregivers, 30 (60%) reported that they could not manage the patients. This reveals the turmoil families undergo when dealing with mentally ill members who become violent; 36 (72%) of the caregivers received help from relatives, neighbours or friends; while only one (2%) received help from police since no one was able to control and was willing to help and people were afraid to come to their house because of the violent behaviour of the patient. About 19 (28%) of the caregivers reported not receiving any help (due to concerns about disclosing mental illness).

One caregiver mentioned, 'My daughter is only 18 years old now; if anyone comes to know about her mental illness, her future will be affected. Sometimes even when she becomes alright people may talk in front of her which might again provoke her mental illness'.

These observations indicate that even though caregivers need help to manage their ill relatives, mental illness still carries stigma and people tend to be afraid about their behavior. Significantly, no caregivers were helped by voluntary health workers (or) social workers during their crises, possibly because their help was not sought because none were available or because caregivers do not see these groups of professionals as trained or willing to help in such situations. This contrasts with the situation in developed countries where the caregiver’s burden is mainly taken care of by the community mental health teams and social services. The majority (N = 30, 60%) did not receive any medical help, although nearly 20 (40%) received help from a medical setup. Among those who received help, 50% were satisfied. These observations indicate even though only 20 (40%) of them that receive help from local physicians, people seek medical facilities for psychiatric problems with some expectation, and once the physician guides them appropriately, they are willing to come for psychiatric treatment. This conforms with the generally observed finding that many people with psychiatric disorders seek help from general physicians before seeking specialist psychiatric help and therefore need appropriate referral.

Most (N = 40, 80%) of the caregivers faced problems in bringing the patient to the hospital. The main problems were economic, distance from a hospital, severe violent behaviour of the patient, the need to use public transport and the unwillingness of the patient to come to the hospital.

Another caregiver said, 'We are poor, my father and mother both are old, and we both are unmarried (patient and caregivers). I do not know where to go and ask for help; my church sister helped us to bring her to the hospital. While coming in the auto, she gave a lot of problems, we were unable to manage her, and she was disturbing the auto driver, dancing, whistling, clapping, talking in foul language'.

These results align with earlier research indicating that poorer mental health is associated with both low socioeconomic status within the family and heightened exposure to social stressors [10]. The majority (N = 42, 84%) of the caregivers expressed that they were very much satisfied with the help provided by the hospital staff independent of their expectations. Caregivers who expressed that they received less help from the staff members reported that they came with a lot of anxiety, physical struggle and tension in bringing the patient to the hospital; they suggested that immediate staff attention and immediate commencement of treatment would reduce their burden [22]. The majority (N = 49, 98%) of the patients were treated within one hour of their arrival. However, one caregiver reported that her relative came with only verbal abuse and became physically violent during the waiting period, and the caregiver suggested that immediate treatment should be provided even if the patient is only verbally abusive to prevent escalation to physical aggression. This suggests the need for effective triage facilities in emergency care.

While caring for patients with violent behaviour, caregivers undergo a lot of emotional stress [12]. The majority (N = 43, 86%) of them said that they were extremely upset and 10% were very upset. Almost all (N = 48, 96%) of the caregivers cried in the presence of the investigator and verbalized their helplessness; 27 (54%) of the caregivers reported being afraid, and 29 (58%) of them were embarrassed about the violent behaviour of the patient. The caregivers were afraid when the patient was a male and young adult, whereas they were more embarrassed with female patients’ disturbed behaviour and foul language (N = 44, 88%); the patient’s violent behaviour was known to others; six (13.63%) of the caregivers did not want to disclose the mental illness. Among them, two (33.33%) were mothers of young girls and four (66.66%) of the patients were known alcoholics. In all, 15 (30%) caregivers said that patients were treated badly by their society, calling them mentally ill in derogatory terms and not treating them like human beings. By contrast, 13 (26%) said that some were treated well and others were treated badly. Almost all the caregivers expressed that if the patient becomes well (N = 33, 66%) and the economic crisis will be solved (18%) and if the patient goes back to work, they would be happy.

The expressed problems of the caregivers had significance concerning their demographic data. There was a significant association between the caregiver’s place of residence and the different needs and problems of the caregivers of patients with violent behaviour. Findings revealed that caregivers from rural areas agreed in high proportion that the mental illness of the patient is known to everyone (97.5%) than those from urban and semi-urban areas (P = 0.001); caregivers from rural areas were more embarrassed (P = 0.023) than others, and rural caregivers were also more afraid (P = 0.012) than others. It therefore appears that caregivers from rural areas are less able to cope with violence in their ill relatives. Regarding the caregivers’ sex, female caregivers did not like to disclose the mental illness, since many of the female caregivers agreed that the mental illness of their patient is not known to others, while the male caregivers were less likely to state that people did not know of the illness in their relatives (P = 0.012). This may relate to differences in perceptions between male and female caregivers, regarding what they feel is known to others and what is not. With the severity of violent behaviour, caregivers were more afraid when the patient was very aggressive (P = 0.013), they were also embarrassed when the severity of aggression was more (P = 0.018). This is also reflected in the opinions of caregivers regarding the use of restraint, where those with severely violent relatives were more likely to endorse the use of restraint even without the consent of the patient or even themselves than caregivers with less aggressive patients. Caring for a loved one with mental illness can be very stressful due to the chronic nature of many mental illnesses [8,21,18]. It can be emotionally and mentally draining when one is a carer for a patient with mental illness [15].

The study's results reveal significant shifts in psychiatric care delivery, presenting both challenges and opportunities for mental health nursing practices in the 21st century. Nursing roles have evolved towards holistic and evidence-based care, emphasizing the involvement of caregivers in patient care planning and intervention. Explanatory model interviews can aid in culturally congruent care planning by assessing caregivers' needs and expectations. Understanding caregivers' perspectives on managing violent patients and addressing their challenges is crucial for enhancing knowledge, attitudes and coping strategies.

Community-based care for mental illness has gained traction, but stigma and fear persist, necessitating education and supportive interventions. Structured teaching programs, counselling and therapy for overwhelmed relatives are lacking and require immediate attention. Recommendations include establishing triage services, allocating time for caregiver input, implementing educational programs, providing guidance and counselling and enhancing public awareness. Further research should focus on replicating the study with larger samples, different language groups and controlled studies to assess intervention effectiveness.

Limitations

This cross-sectional study spanned only eight weeks, potentially limiting its ability to capture the evolving perspectives of caregivers over time. In addition, it was conducted at a single centre, offering only a snapshot of the challenges caregivers may face elsewhere. The study's reliance on qualitative methods means that its findings are subject to the subjective expressions of caregivers.

## Conclusions

This study offers a comprehensive insight into caregivers' needs and problems. Through their expressed verbatim, we gained valuable insights into the fears, expectations and difficulties they encounter in understanding and managing psychiatric patients, particularly those exhibiting violent behaviour. The responsibility of caring for mentally ill individuals with violent tendencies imposes a significant burden on caregivers. However, fostering strong interpersonal relationships and maintaining open communication with healthcare professionals can provide guidance and support, significantly aiding caregivers in managing this burden and improving their ability to deliver effective care.

Moreover, conducting public campaigns and providing personal counselling, increasing caregiver awareness and fostering a clear understanding of mental illness and the management of violent behaviour could result in improved treatment outcomes, prognosis and patient rehabilitation. Consequently, this could lower the probability of recurrence.

## Appendices

                                                                                **        Part I**

                                                                           Demographic Data

                                                                (Patients/Caregivers/Clinical data)

                   Date:                                         Time:                                              Hospital Number

 Patient Data:

1.      Age

2.      Gender

3.      Marital status

4.      Education

5.      Occupation

6.      Monthly income

7.      Religion

8.      Working status? Yes/ No

Caregiver Data:

9.      Relationship of the primary informant

10.    Age

11.     Gender

12.    Marital Status

13.    Education

14.    Occupation

15.    Place: urban/semi-urban/rural

16.    Distance from Hospital(km)

17.    Mode of transport

Clinical data:

18.    Were patient restraints at arrival?  Yes/No. If yes, describe

19.    Number of other attendants with patient

20.    Relationship with patient

21.    Any injury to the patient? Yes/No If yes, describe

22.    Any injury to relatives? Yes/No. If yes, describe

23.    Any emergency treatment in the previous week? Yes/No. If yes, describe

24.    Diagnosis

25.    Duration of current episode of illness (days)

26.    First episode?

27.     Any other associated illness Yes/No

28.    Severity of illness - CGI Score

29.    Severity of violent behaviour- OAS Score

                                                           ** Part II**

                                       Clinical Global Impression (CGI)

Reference: Guy W, editor. ECDEU Assessment Manual for Psychopharmacology. 1976. Rockville, MD, U.S. Department of Health, Education, and Welfare

**Rating:**  Clinician-rated

**Instruction:** Indicate only one response for each question by placing a cross(x) in the appropriate number box.

Severity of Illness:

Consider your total Clinical Experience with this patient population, how mentally ill is the subject at this time?

Normal not at all ill                        1

Borderline mentally ill                    2

Mildly mentally ill                           3

Moderately mentally ill                  4

Markedly mentally ill                      5

Severely mentally ill                       6

Among the most extremely ill        7

                                                                  **Part -III      **

                                                      Overt Aggression Scale (OAS)

Reference: Yudofsky SC, Silver JM, Jackson W, et al.: The Overt Aggression Scale for the objective rating of verbal and physical aggression. American Journal of Psychiatry. 1986, 143:35-39. 10.1176/ajp.143.1.35

I**nstructions**: Please place an X on the right-hand margin, and check if they are present.

**Aggressive Behaviors (check all that apply)    **      

**Verbal Aggression**    

-          Makes loud noises, shouts angrily     

-          Yells mild personal insults, e.g. "You’re stupid."     

-          Curses viciously uses foul language in anger and makes moderate threats to others or self.           

-          Makes clear threats of violence towards others or self (‘I’m going to kill you", or "I may just kill myself.)          

**Physical Aggression Against Objects**         

-          Slams the door, scatters clothing makes a mess.       

-          Throws objects down, and kicks furniture without scratching it or making marks in the wall.        

-          Breaks objects, kicks in walls, smashes windows.    

-          Sets fires, throws objects dangerously

**Physical Aggression Against Self  ** 

-          Picks or scratches skin hits self, pulls hair (with no or minor injury only).  

-          Bangs head, hits fist into objects, throws self onto the floor or into objects (Hurst self without serious injury).

-          Small cuts or bruises, minor burns.   

-          Mutilates self, causes deep cuts, bites that bleed, internal injury, fracture, loss of consciousness, and loss of teeth.   

Physical Aggression Against Other People

-          Makes threatening gestures, swings at people, grabs at clothes.       

-          Strikes, kicks, pushes and pulls hair (without injury to them).        

-          Attacks others, causing mild to moderate physical injury (bruises, sprain welts).   

-          Attacks others, causing severe physical injury (broken bones, deep lacerations, internal injury)    

                                                                                **   Part -IV**

                                                                                 NEEDS AND PROBLEMS

The following questions relate to the problems you faced with your relative and your needs and expectations when you brought him/her to the hospital. We hope that your answers will help us understand the problems you face and your needs better so that we can improve the care that we provide

1.       What made you bring your relative to the hospital today?

2.       How long has your relative been showing violent behaviour?  Describe the types of violent behaviour that were shown (Verbal abuse/threats, physical aggression, destruction of property, etc.)

3.       How were you managing the patient when he/she is violent? Did you or others shout/ beat/restrain your relative?

4.       Did you have help in managing your relative?

5.       What difficulties did you face in bringing your relative to the hospital?

6.       What were your expectations from the hospital staff when you came to this hospital?

7.       How long did you have to wait before a hospital staff member attended to you?

8.       Can you rate how upset you are by your relative’s behaviour on the following scale?                                                      1. Not upset; 2. Somewhat upset; 3. Upset; 4. Very upset; 5. Extremely upset

9.       Are you/were you afraid of your relative when he/she was violent?

What frightened you the most?

Did you fear for your safety/life?

Were you embarrassed by your relative’s mental illness?

10.   Is there anything else that you would like to share about your relative or your reactions to his/her illness or your needs?
